# Veterans Affairs databases are accurate for gout-related health care utilization: a validation study

**DOI:** 10.1186/ar4425

**Published:** 2013-12-31

**Authors:** Jasvinder A Singh

**Affiliations:** 1 Medicine Service and Center for Surgical Medical Acute Care Research and Transitions (C-SMART), Birmingham VA Medical Center, Birmingham, AL, USA; 2 Department of Medicine at School of Medicine, and Division of Epidemiology at School of Public Health, University of Alabama at Birmingham, 510 20th Street South, Faculty office tower 805B, Birmingham, AL 35294, USA; 3 Department of Orthopedic Surgery, Mayo Clinic College of Medicine, Rochester, MN, USA

## Abstract

**Introduction:**

The aim of this study was to assess the accuracy of Veterans Affairs (VA) databases for gout-related health care utilization.

**Methods:**

This retrospective study utilized VA administrative and clinical databases. A random sample of gout patients with visits (outpatient, inpatient or emergent/urgent care) with or without the diagnosis of gout (International Classification of Diseases, ninth revision, common modification ICD-9-CM code of 274.x or 274.xx) at the Birmingham VA hospital was selected. A blinded abstractor performed a review of VA electronic health records for the documentation of gout or gout-related terms (gouty arthritis, tophaceous gout, tophus/tophi, acute gout, chronic gout, podagra, urate stones, urate or uric acid crystals and so on) in the chief complaint, history of present illness or assessment and plan for the visit; this constituted the gold standard for gout-related utilization. The accuracy of database-derived gout-related claims was assessed by calculating sensitivity, specificity, and positive and negative predictive values (PPV and NPV).

**Results:**

Of 108 potential visits, 85 outpatient, inpatient or urgent care/emergency room visits to a health care provider (85 patients: 84 men and 1 woman with a mean age of 63 years) and retrievable data from medical records constituted the analyzed dataset. Administrative claims for gout-related utilization with ICD-9 code for gout were accurate with a PPV of 86%, specificity of 95%, sensitivity of 86% and NPV of 95%.

**Conclusions:**

VA databases are accurate for gout-related visits. These findings support their use for studies of health services and outcome studies. It remains to be seen if these findings are generalizable to other settings and databases.

## Introduction

Gout is the most common inflammatory arthritis in adults [1]. It has a significant effect on patients’ quality of life, productivity, health care costs and utilization [2-4]. In the era of comparative effectiveness research (CER) [5], comparisons of various treatments with regard to health care utilization and costs are very important from patient, provider and policy maker perspectives. As new and more expensive gout treatments become available and the cost of gout treatment increases, a critical question is whether the higher costs of treatment are counterbalanced by more benefits to the patient, in terms of reduced morbidity and reduced gout-related health care utilization. Thus, it is important to assess if large administrative databases can provide valid health care utilization and outcomes data for CER in gout.

Two studies assessed the accuracy of administrative claims/codes for gout [6,7]. A study in a managed care plan found that an International Classification of Disease ninth revision, common modification (ICD-9-CM) code for gout had a positive predictive value of 61% compared to rheumatologist adjudication of medical record abstractions [7]. The second study found that the concordance of ICD-9-CM code from databases with gout classification criteria was low at 30% to 36% [8]. The wide variation of accuracy of administrative claims/codes may be attributable to differences in patient populations and the gold standards between these studies, and raises the question whether the use of administrative databases is a valid approach for health services research in gout.

It is critical to establish the accuracy of administrative database definitions for a diagnosis of gout (including ICD-9-CM codes) and for gout-related utilization before we can rely on the findings from health services and outcomes studies in gout. Several studies of gout-related health care utilization from large databases have been published recently [9-11]. In contrast, to the best of our knowledge there are no published validation studies assessing the accuracy of gout-related health care utilization. Thus, it is unknown whether the administrative-derived data for gout-related visits are accurate and specific for gout-related utilization. The Veterans Affairs (VA) is the largest integrated health care system in the US. It serves more than five million veterans annually [12]. The objective of this study was to assess the accuracy of gout-related visits in the VA administrative and claims databases.

## Methods

### Cohort selection

For this validation study, a retrospective cohort of veterans with a known prior diagnosis of gout with any health care encounter at Birmingham VA Medical Center in the fiscal year 2006 (October 2005 to September 2006) was selected. Fiscal year 2006 was chosen for this study for the following reasons: (1) it allowed an adequate follow-up duration for the included patients; (2) there were no ongoing or new quality initiatives in gout or other arthritic conditions at Birmingham VA medical center; (3) no new treatment guidelines were published; and (4) no new gout drugs were launched. A data programmer created a dataset of two samples that were mixed randomly: VA health care visits with or without gout (based on the presence of ICD-9-CM code of 274.x or 274.xx) as the primary or secondary diagnosis for the visit other diseases included hyperuricemia without gout. The Institutional Review Board at Birmingham VA Medical Center approved the study and waived the requirement for patient consent for this retrospective database study.

### VA databases

The random sample was drawn from the administrative data available from the Austin Automation Center, a national VA data warehouse for the Birmingham VA cohort. These national computerized datasets have detailed data on VA health care provided to enrolled veterans, allowing the assessment of demographics and health care utilization. Data included the date of the visit, type of visit (outpatient, inpatient and emergent/urgent care), primary diagnosis (the main reason for health care visit), up to 15 secondary diagnoses and patient demographics including patient age and gender.

A standardized data extraction form was used to abstract data for each visit identified from the administrative databases from the computerized medical records of the patients. Data were abstracted related to the presence or absence of gout or gout-related complaints in the various parts of the clinical note (chief complaint, history of present illness and assessment and plan), gout-specific medications from the pharmacy medication list in the note (allopurinol, probenecid, colchicine) and results of imaging studies, including X-rays, and laboratory tests (serum uric acid, crystal analyses in synovial fluid or tophus).

### Definitions of gold standard and test standard

The gold standard for this study was whether the visit was gout-related as determined by the review of the medical records. Gout-related visit was defined *a priori* as the mention of gout or gout-related terms (gouty arthritis, tophaceous gout, tophus/tophi, acute gout, chronic gout, podagra, urate stones, urate or uric acid crystals) in the chief complaint, history of the present illness or assessment and plan for the index visit in the medical records. This indicated that gout was the main reason or one of the main reasons for the index visit. The test standard was the presence of an ICD-9-CM code for gout (274.x or 274.xx) in VA inpatient or outpatient databases for the index patient visit. The author, a senior epidemiologist, experienced in data abstraction [13,14] and blinded to the test standard (that is, gout diagnosis for the visit from VA databases), abstracted the data from the VA electronic health records. He considered limiting the study cohort to only those gout patients who have demonstration of urate crystals in joint/bursa fluid or mass, but decided to look for all patients with gout, given that <5% of patients with gout have crystal-proven gout [15]. When a visit listed in the databases was missing from the medical records, the abstractor reviewed notes in the immediate period (before and after) to assess if the visit date was miscoded.

### Statistical analyses

The administrative data definition of gout-related visit, that is, gout as the primary or secondary diagnosis in outpatient, inpatient or emergent/urgent care setting in the VA administrative database (test standard), was compared to the gold standard of medical record documentation that the patient’s visit was related to gout. Sensitivity, specificity, positive and negative predictive value (PPV and NPV) and kappa statistic for administrative data were calculated, in a similar manner as that previously reported for rheumatoid arthritis and spondylarthritides [14,15]. Sensitivity was the fraction of those with a gout-related visit according to the gold standard that were correctly identified as positive by the VA database definition. Specificity was the fraction of visits not for gout according to the gold standard that were correctly identified as negative by the database definition. PPV was the proportion of those with gout-related visits in the VA database who met the gold standard definition of medical chart documentation. NPV was the proportion of visits not for gout in the VA database that did not meet the gold standard definition. The kappa coefficient was used to describe agreement (beyond chance) between the medical record documentation of gout (gold standard) and VA database documentation (test standard).

### IRB approval

The Institutional Review Board (IRB) at the Birmingham VA Medical Center approved this study. The need for patient consent was waived by the IRB, since this was a retrospective database study. All investigations were conducted in conformity with ethical principles of research.

## Results

Of the 108 potential visits of gout patients, 85 visits (85 patients: 84 men, 1 woman; mean age, 63 years) with a medical record note constituted the main analyzed dataset. The 23 visits excluded from the main analysis were either laboratory or telephone encounters only (n = 15) or visits recorded in VA databases for which no documentation of a visit (n = 8) was found in medical records; they were included in sensitivity analyses.

According to the gold standard of chart documentation, 21 visits were related to gout and 64 were not (Table 1). There were three visits coded as gout-related visits in databases that did not have medical record documentation as related to gout: one visit each to discuss blood pressure medication, regular follow-up for multiple medical problems, and for increased blood sugar. Three visits coded as not related to gout in administrative databases were related to gout based on medical record documentation: one patient each with continuing acute gout flare, a new diagnosis with documentation of urate crystals in knee joint fluid and chronic gout stable on allopurinol (Table 1).

**Table 1:** Accuracy of VA database for gout-related visits

	**True**	**True**	**False**	**False**	**Sensitivity**	**Specificity**	**PPV**	**NPV**	**Kappa**
	**Positive**	**Negative**	**Positive**	**Negative**	**(95% CI)**	**(95% CI)**	**(95% CI)**	**(95% CI)**	**(95% CI)**
	**(n/N)**	**(n/N)**	**(n/N)**	**(n/N)**					
**Main analyses (number = 85), including only patients with outpatient, inpatient, ER or Urgent care visits**									
All visits (number = 85)	18/85	61/85	3/85	3/85	0.86	0.95	0.86	0.95	0.81
					(0.78, 0.93)	(0.91, 1.00)	(0.78, 0.93)	(0.91, 1.00)	(0.66, 0.96)
Outpatient visits only (number = 76)	16/76	55/76	3/76	2/76	0.89	0.95	0.84	0.96	0.82
					(0.82, 0.96)	(0.90, 0.99)	(0.76, 0.92)	(0.93, 1.00)	(0.67, 0.97)
Inpatient, emergent or urgent care visits only (number = 9)	2/9	6/9	0/9	1/9	0.67	1.00	1.00	0.86	0.73
					(0.57, 0.77)	(N/A)	(N/A)	(0.78, 0.93)	(0.24, 1.00)
**Sensitivity analyses (number = 108), including all visits in database**^**a**^									
All visits (number = 108)	19/108	79/108	6/108	4/108	0.83	0.93	0.76	0.95	0.73
					(0.75, 0.90)	(0.88, 0.98)	(0.68, 0.84)	(0.91, 0.99)	(0.57, 0.89)
Outpatient visits only (number = 99)	17/99	73/99	6/99	3/99	0.85	0.92	0.74	0.96	0.73
					(0.78, 0.92)	(0.87, 0.97)	(0.66, 0.82)	(0.92, 1.00)	(0.57, 0.90)
Inpatient, emergent or urgent care visits only (number = 9)	2/9	6/9	0/9	1/9	0.67	1.00	1.00	0.86	0.73
					(0.57, 0.77)	(N/A)	(N/A)	(0.78, 0.93)	(0.24, 1.00)

^a^These included telephone and laboratory visits as well as those for which there was no documentation in the medical records. 95% CI, 95% confidence interval; N/A, not applicable; NPV, negative predictive value; PPV, positive predictive value;

The VA database claims for gout-related visits had moderate to high specificity of 95.3%, PPV of 85.7% and sensitivity of 85.7% (Table 1). When the analyses were limited to outpatient claims only, specificity was still high at 94.8%, PPV was moderate at 84.2% and sensitivity was high at 88.9%. For non-outpatient claims (inpatient, emergent or urgent care), specificity and PPV were 100% each and sensitivity was 66.7%.

Sensitivity analyses that included the entire cohort (n = 108; including telephone and laboratory visits and those with no note) revealed that specificity of VA claims was high at 92.9%, PPV was moderate at 76% and sensitivity was 82.6%. Sensitivity analyses for the outpatient and non-outpatient claims for the entire cohort showed minimal differences compared to the main analysis (Table 1).

## Discussion

In this validation study, VA database claims for gout-related visits based on ICD-9-CM codes have moderate to high specificity and PPV meaning that most gout-related visits identified by VA administrative databases were, in fact, related to gout. Specificity and PPV for non-outpatient claims were numerically higher and sensitivity lower, compared to outpatient claims. In the absence of previous studies, these findings are novel and have important implications for health services studies using administrative databases. This study shows that if one were to use a broad approach of using any VA administrative claims, 76% of all visits coded as related to gout in VA administrative and clinical databases would truly be related to gout (PPV). If one takes a more sophisticated approach of excluding telephone encounters or visits with laboratory test only as non-specific, which can be easily done in most administrative databases including the VA, the specificity exceeded 95% and PPV exceeded 85%. Thus, VA databases were accurate for identifying gout-related utilization. The accuracy further improved by excluding visits for laboratory tests only and telephone encounters.

These predictive values are acceptable for health services studies, since the diagnostic accuracy is similar to some of the most commonly used laboratory/diagnostic tests in medicine. Our accuracy statistics cannot be directly compared to validation studies by Harrold and Malik *et al*. due to the differences in the gold standard and the study question (accuracy of gout diagnosis compared to accuracy of gout-related health care visit) [6,7]. Similar to our previous validation studies in other types of arthritis [13,14], this study is an important step in developing validated outcomes for health services and outcomes research that rely heavily on databases. Health services and CER studies often rely on large databases to address policy-level questions. Administrative databases such as Medicare, health plans, VA and so on are often used for the comparison of the effectiveness of various medications, treatment approaches or systems interventions to improve disease outcomes and reduce health care utilization. An inherent assumption made in these studies is that databases are accurate for health services utilization, which may not always be true. This study validates this assumption for gout-related utilization in the largest integrated health care system in the US, that is, the VA, that serves more than five million US veterans and has state-of-the-art sophisticated electronic health care record systems and national datasets designed for research [12]. One must consider that, although VA is a national health care system with uniform access, benefits and staffing, and there are no obvious known systematic differences in patients or patient care compared to other facilities, this was not a random sample of all VA patients. Therefore, a further validation of these findings in a national sample will strengthen these conclusions.

Specificity and PPV of non-outpatient visits for gout (inpatient, emergent or urgent care) was even higher than that noted for outpatient visits. In fact, the databases are very accurate for non-outpatient gout visits with 100% specificity and 100% PPV, although sensitivity was lower. This has important implications. A PPV of 100% indicates that one can use gout-related inpatient, emergent or urgent care visits in VA databases with great confidence in health services and outcomes research. This easier approach is a more valid alternative to the unvalidated administrative definitions for gout flares used previously [16-19]. No validation was done except in one study, that mentioned performing a chart review on 100 patients, but did not provide any accuracy statistics [18]. A better accuracy of databases for non-outpatient visits may indicate the focused nature of non-outpatient visits that leads to more accurate coding, as opposed to routine care for multiple medical conditions during scheduled outpatient visits.

This study has several limitations. The findings may not be generalizable to other health care settings (health maintenance organizations) and datasets (Medicare) and/or to other musculoskeletal conditions. It remains to be seen whether this approach can be validated in other large databases; studies are needed to examine the validity of this approach. Under-documentation in medical records is well known and, therefore, some visits deemed as not related to gout based on the medical record gold standard may have been labeled incorrectly. Considering this possibility, broad criteria for a visit to be determined as related to gout were made, namely, the presence of gout or gout-related complaints in any part of the provider’s note. A limitation to bear in mind is that while common gout-related terms including the presence of urate crystals were searched, accuracy in patients with crystal-proven gout was not tested, since a very small proportion of patients with gout have crystal-proven gout. It is possible that a greater health care provider understanding or awareness of gout may influence the extent of medical record documentation of gout, an important determinant that cannot be assessed with the current study. This is a question of interest for future studies and will be particularly relevant where the accuracy statistics are low or not acceptable.

## Conclusion

In conclusion, the VA databases were found to be accurate for gout-related visits. The accuracy was higher for inpatient, emergent or urgent care visits, compared to outpatient visits related to gout. This implies that VA administrative databases can be used for health services outcomes research and CER in gout.

## Abbreviations

CER: Comparative effectiveness research; ICD-9-CM: International Classification of Disease ninth revision common modification; NPV: Negative predictive value; PPV: Positive predictive value; VA: Veterans Affairs

## Competing interests

Dr. Singh has received research grants from Takeda and Savient and consultant fees from Takeda, Savient, Regeneron and Allergan. Dr. Singh is a member of the executive of OMERACT, an organization that develops outcome measures in rheumatology, and receives arms-length funding from 36 companies. Dr. Singh is also a member of the American College of Rheumatology's Guidelines Subcommittee of the Quality of Care Committee and Veterans Affairs Rheumatology Field Advisory Committee.
